# Supplementary material for: Sound Localization and Separation in 3D Space Using a Single Microphone with a Metamaterial Enclosure
Source: Adv Sci (Weinh). 2019 Dec 27;7(3):1902271. doi: 10.1002/advs.201902271 (PMC7001621; doi:10.1002/advs.201902271)
Supplement: Supplementary file 1 — Supporting Information [file ADVS-7-1902271-s001.pdf]

Copyright WILEY-VCH Verlag GmbH & Co. KGaA, 69469 Weinheim, Germany, 2019.

ADVANCED SCIENCE
Open Access

Supporting Information

for *Adv. Sci.,* DOI: 10.1002/advs.201902271

Sound Localization and Separation in 3D Space Using a Single Microphone with a Metamaterial Enclosure

Xuecong Sun, Han Jia, Zhe Zhang, Yuzhen Yang, Zhaoyong Sun, and Jun Yang**

Supporting Information for

Sound Localization and Separation Using a Single Microphone with a Metamaterial Enclosure

Xuecong Sun[1,2], Han Jia[1,2,3*], Zhe Zhang[1,2], Yuzhen Yang[1,2], Zhaoyong Sun[1], and Jun Yang[1,2,3*]

S1. The Simplified Effective Model of the Acoustic Channel Module

As mentioned in the main text, the geometric parameters of the acoustic channel module (ACM), including volume of the cavities, filling ratio and location distribution of the holes, would directly affect the frequency response. In order to demonstrate the relationship between the frequency response and the geometric parameters, a simplified model of the ACM is established which is shown in **Figure S1**(a). The model includes three layers of square plates and two cuboid cavities. The frequency dependent transmission characteristic from the incident plane to the exit plane can be expressed as:

$$T(\omega) = M_1(\omega) N_1(\omega) M_2(\omega) N_2(\omega) M_3(\omega) \tag{1}$$

Here, $M_1(\omega)$, $M_2(\omega)$ and $M_3(\omega)$ are the frequency response matrices of the three perforated plates respectively; $N_1(\omega)$ and $N_2(\omega)$ are the frequency response matrices of two cavities respectively. The frequency response matrices change with the geometric sizes and distributions of the ACMs, including the volume of the cavities, the filling ratio, and the location distribution of the holes. Three groups of simulations were designed in order to assess the effect of these factors on the frequency response of the ACM. In each group of simulations, only one factor was changed while the other two factors were kept constant. The top view of the perforated plates corresponding to each simulation are shown in Figure S1(b), **Figure**

S2(a) and **Figure S3**(a), respectively. The simulated frequency responses are shown in Figure S1(c), Figure S2(b) and Figure S3(b) respectively. The simulation results show that the volume of the cavities and the filling ratio affect the frequency response in both the low and high frequency regions, while the location distribution of the perforated holes is more capable of affecting the high-frequency response. In general, the different frequency responses are contributed by ACMs with different geometric sizes and distributions. So by changing these factors to construct different ACMs in different directions, the frequency response of the ME would be directionally dependent.

S2. Construction of the measurement matrix A.

- The matrix $\mathbf{A} = [a_1, a_2, \dots, a_Q]$ can be regarded as a dictionary. The number of columns of the matrix $\mathbf{A}$ is $Q = n \times m$, where n is the number of all the potential sound sources in 3D space and m is the size of the audio library. The lth column a_l is a P-length column vector and can be expressed as:

$$\begin{aligned} a_l &= \mathrm{VSPCA}\{h_i \circ f_j\}, \\ l &= (i-1)m + j, \end{aligned} \tag{2}$$

Here, f_j is the spectral amplitude vector of the jth audio in the audio library $\mathbf{F} = [f_1, f_2, \dots, f_m]$; h_i is the ith directional frequency response amplitude vector of the MSLS $\mathbf{H} = [h_1, h_2, \dots, h_n]$. The operational symbol "∘" is the Hadamard product, which means element-wise multiplication rather than matrix multiplication.[1] Thus, the $h_i \circ f_j$ can be obtained by traversing all of the locations with each of the audios and calculating the spectrum amplitude of the measuring signals. Then, the samples

$h_i \circ f_j$ are projected to the direction of maximum variance through VSPCA, which is used to build the matrix **A**. Similarly, the observation vector y can be expressed as

$$y = \mathrm{VSPCA}\{y_0\}, \tag{3}$$

where y_0 is the spectral amplitude of the test signal collected by the microphone. Details concerning the VSPCA algorithm can be found in the Supplementary Note 3.

S3. Variable Sparsity Principal Component Analysis (VSPCA)

Based on traditional principal component analysis (PCA) and the training set, we can calculate the transform matrix **W**, the mean values $\boldsymbol{\mu} = [\mu_1, \mu_2, \ldots, \mu_{d_1}]$ and the standard deviation $\boldsymbol{\sigma} = [\sigma_1, \sigma_2, \ldots, \sigma_{d_1}]$ of the characteristics.[2] The original training samples can be expressed as $x_{train} = [x_{train}^1,\ x_{train}^2, \ldots, x_{train}^{d_1}]^T$. Here, d_1 is the dimensions of the original samples in the training set. Then, the dimension of the original sample $U = [u_1,\ u_2, \ldots, u_{d_1}]^T$ can be reduced, which can be expressed as

$$\begin{aligned} \bar{u}_i &= \frac{u_i - k\mu_i}{\sigma_i} \quad (i = 1, 2, \ldots, d_1), \\ v_j &= \mathbf{W}(:, \mathrm{j})^T \bar{U} \quad (j = 1, 2, \ldots, d_2), \end{aligned} \tag{4}$$

where $\bar{U} = [\bar{u}_1,\ \bar{u}_2, \ldots, \bar{u}_{d_1}]^T$ represents a normalized sample, $\mathbf{W}(:, \mathrm{j})^T$ is the jth column of the transform matrix **W**, and k is the sparsity of the sample U. Thus, the sample after the dimension reduction can be expressed as $V = [v_1,\ v_2, \ldots, v_{d_2}]^T (d_2 < d_1)$. When sparsity k equals one, the VSPCA becomes the traditional PCA. The training samples in our listening tests only contain conditions in which there is one activated source in the 3D space (thus k=1), while the testing samples contain conditions in which there is more than one activated source. A k-sparse sample can be regarded as the simple superposition of certain k one-sparse

samples. In order to make sure that all of the samples can be mapped to the same space, we need to introduce the parameter k in the process of normalization.

S4. Supplementary Experiment in pitching direction

In this case, the system was deployed vertically on the table and speakers were placed at the same level, as shown in **Figure S4**(a). The audio library used in the tests contained 6 typical sound signals often heard in the street, which are shown in **Figure S5**. The reconstruction results are shown in Figure S4(b), which further prove the spatial resolution of the MSLS in pitching direction.

Supporting Figures

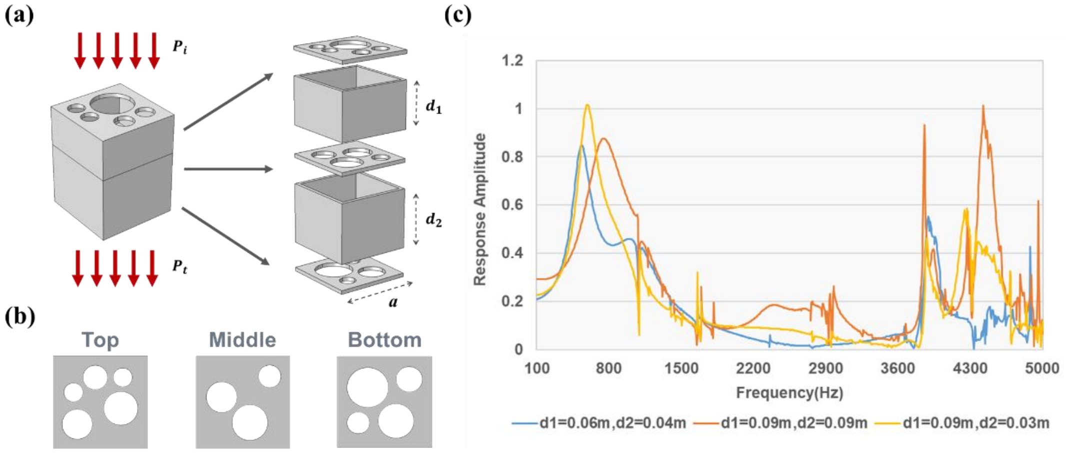

Figure S1. Effective model and frequency responses of the ACMs with different volume cavities. (a) An effective model of the ACM, where p_i is the incident wave and the p_t is the transmission wave. The side length of the cavities is a fixed value, a=0.1m. The volume of the cavities can be adjusted by changing the height of the cavities d_1 and d_2. (b) Top view of the perforated plates: Top plate, middle plate and bottom plate. (c) Frequency responses of ACM with different volume cavities.

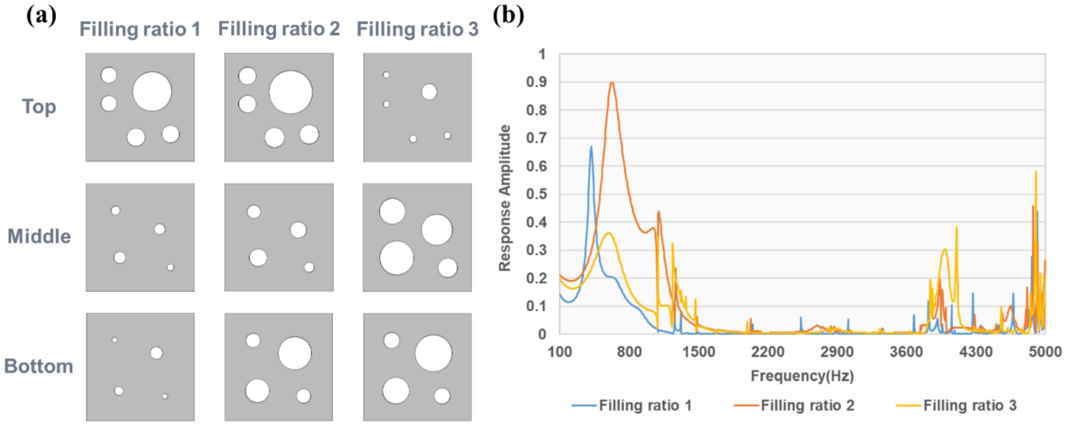

Figure S2. Frequency responses of the ACMs with different filling ratios. (a) Top view of the perforated plates in three different designs of the ACMs, where the filling ratio of the perforated plates is varied to create different frequency responses. For the three modules, the positions of the holes are fixed, while the radii are varied. And the size of the two cavities is also fixed: $a = 0.1$m, $d_1 = 0.05m$ and $d_2 = 0.08m$. (b) Frequency responses of the three different ACMs.

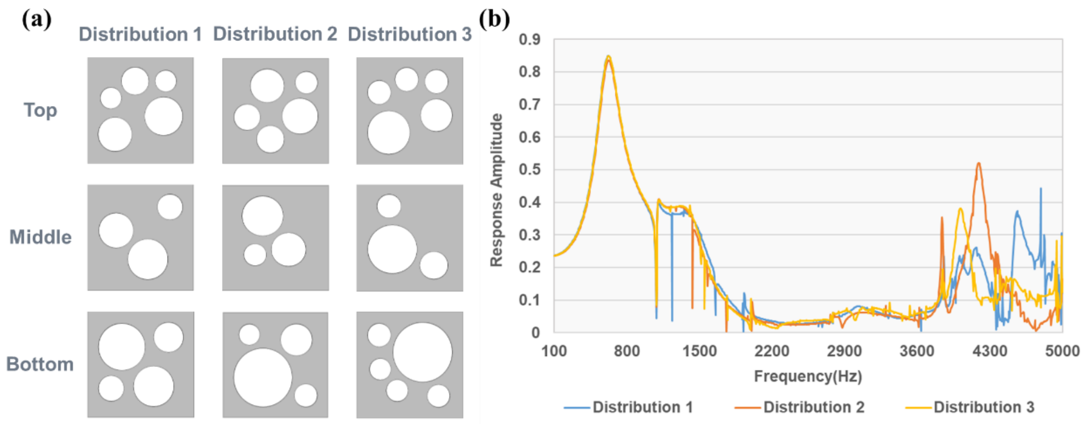

Figure S3. Frequency responses of the ACMs with different distribution of holes. (a) Top view of the perforated plates in three different designs of the ACMs, where the distribution of holes is varied to create different frequency responses. In the three ACMs, the porosity of the perforated plates is fixed: $w_1 = 0.32$, $w_2 = 0.25$ and $w_3 = 0.4$. And the size of the two cavities is also fixed: $\mathrm{a} = 0.1\mathrm{m}$, $d_1 = 0.05m$ and $d_2 = 0.08m$. (b) The corresponding frequency responses of the three different ACMs.

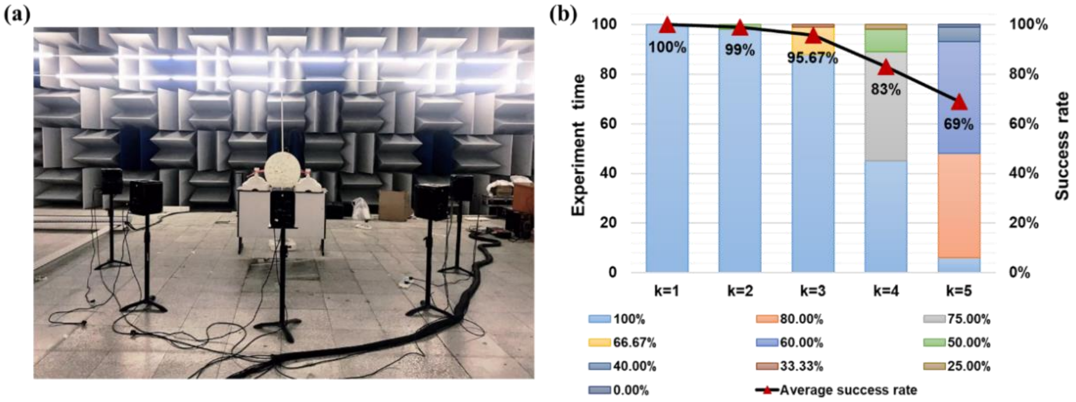

Figure S4. Pitching direction Experiment and results. (a) Measurement performed in pitching direction (b) The results of the measurement.

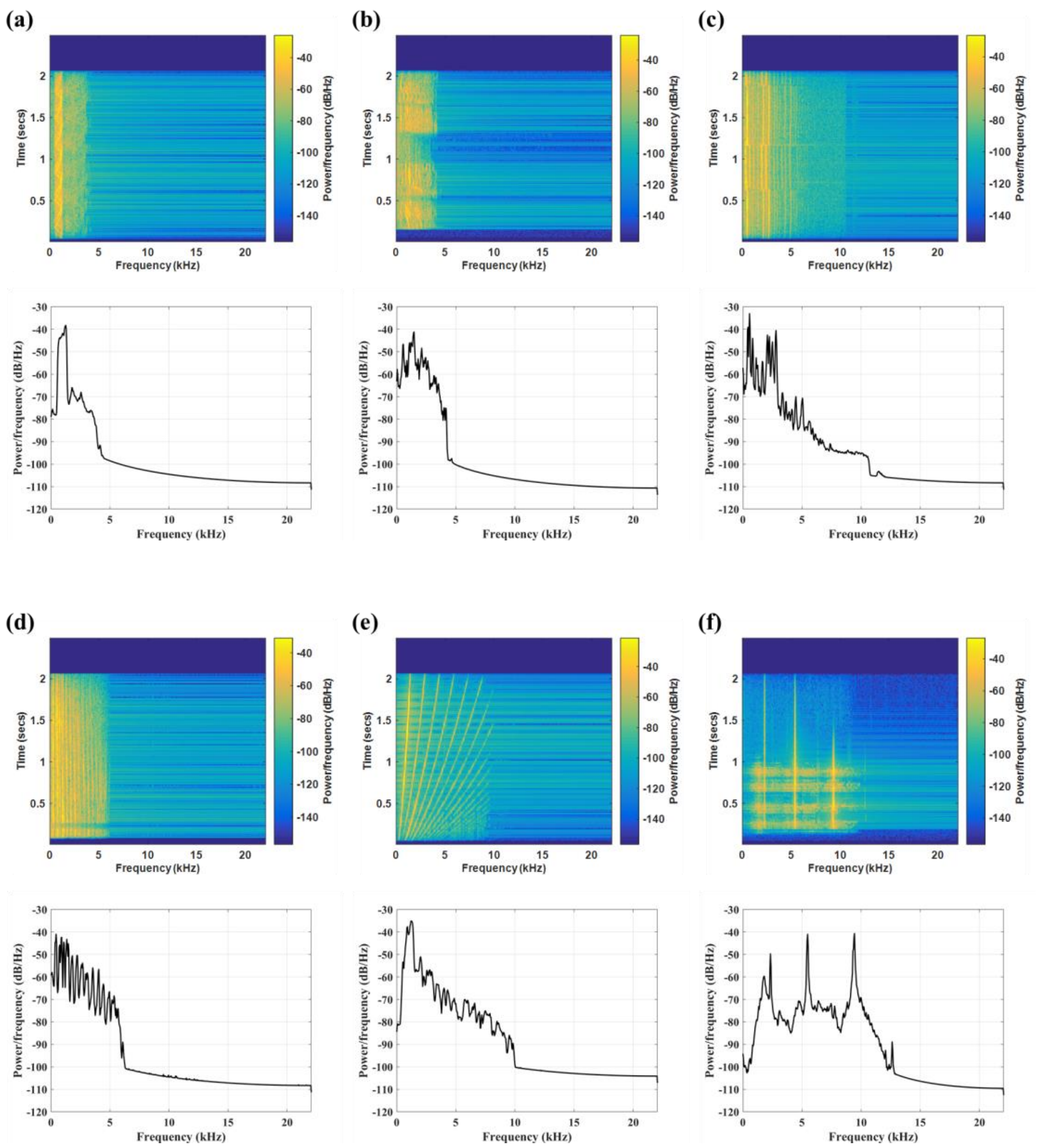

Figure S5. Spectrogram (top) and power spectral density (bottom) of six types of signals often heard in the street, including (a) police car, (b) backing car, (c) ambulance, (d) car whistle, (e) fire engine and (f) bicycle bell.

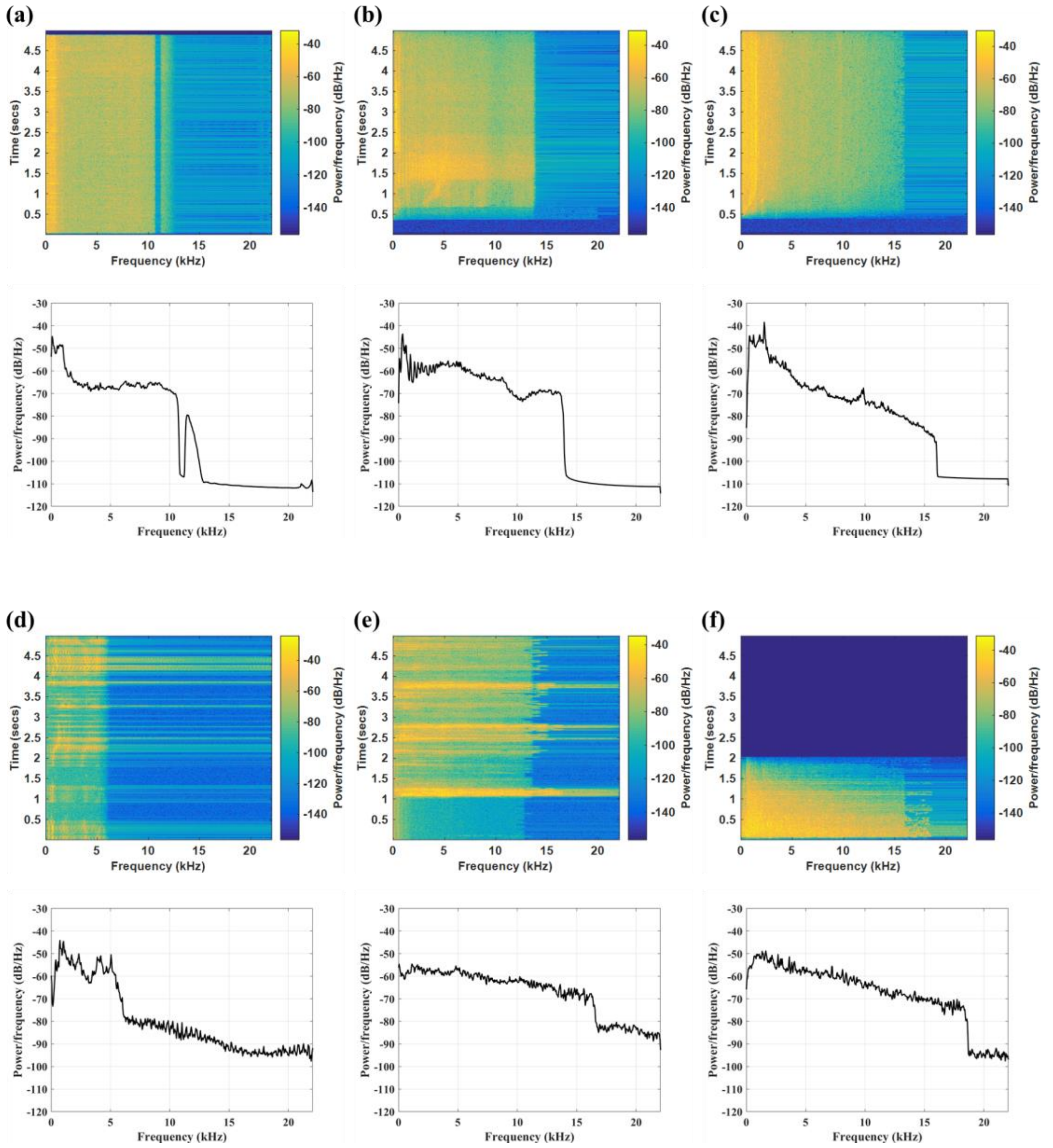

Figure S6. Spectrogram (top) and power spectral density (bottom) of six types of signals often heard in the home, including (a) cooking, (b) flushing the toilet, (c) drying hair, (d) laughter, (e) tearing up paper and (f) something breaking.

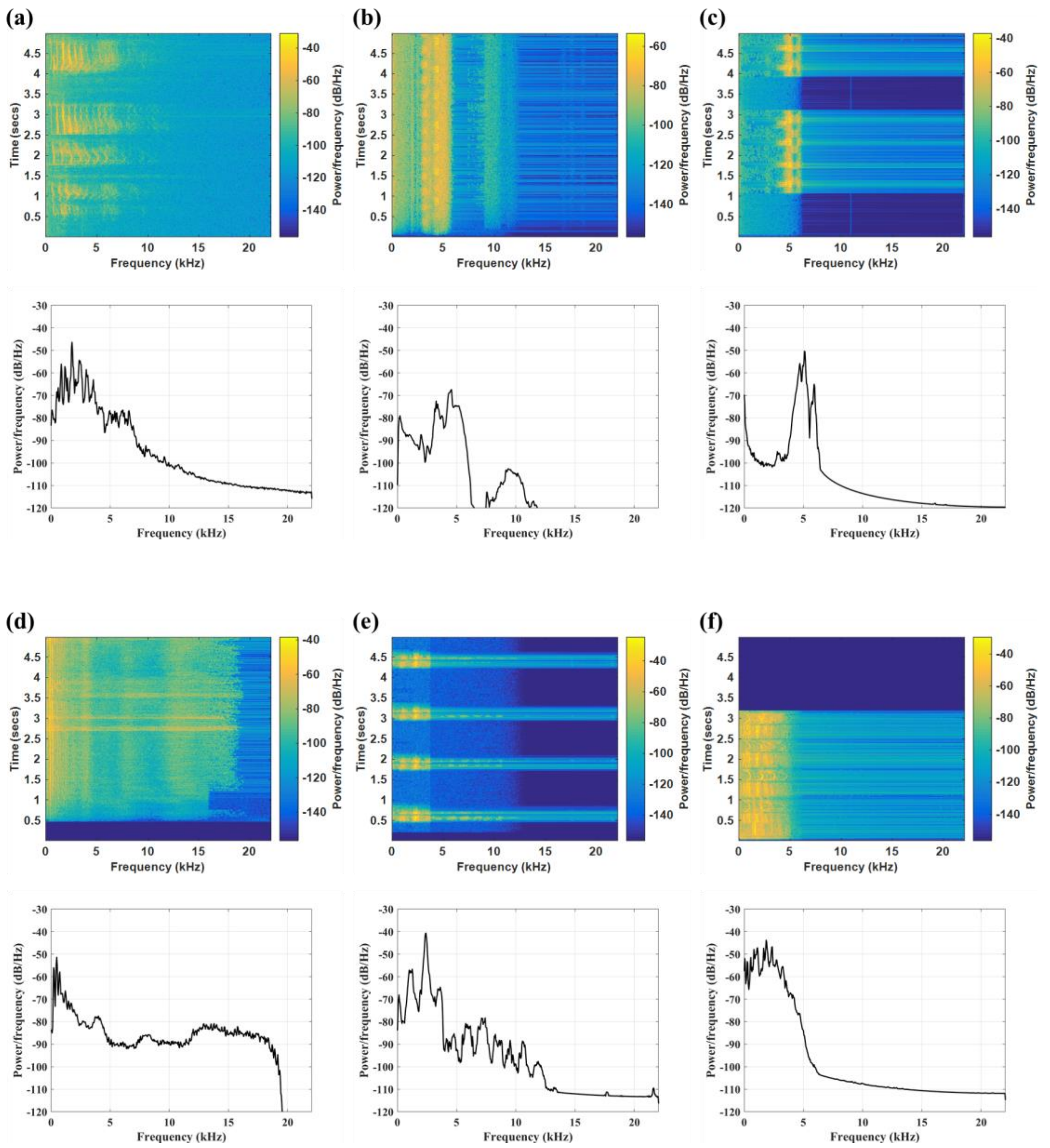

Figure S7. Spectrogram (top) and power spectral density (bottom) of six types of signals often heard in the animal farm, including (a) cat, (b) cicada, (c) cricket, (d)bee, (e) frog and (f) duck.

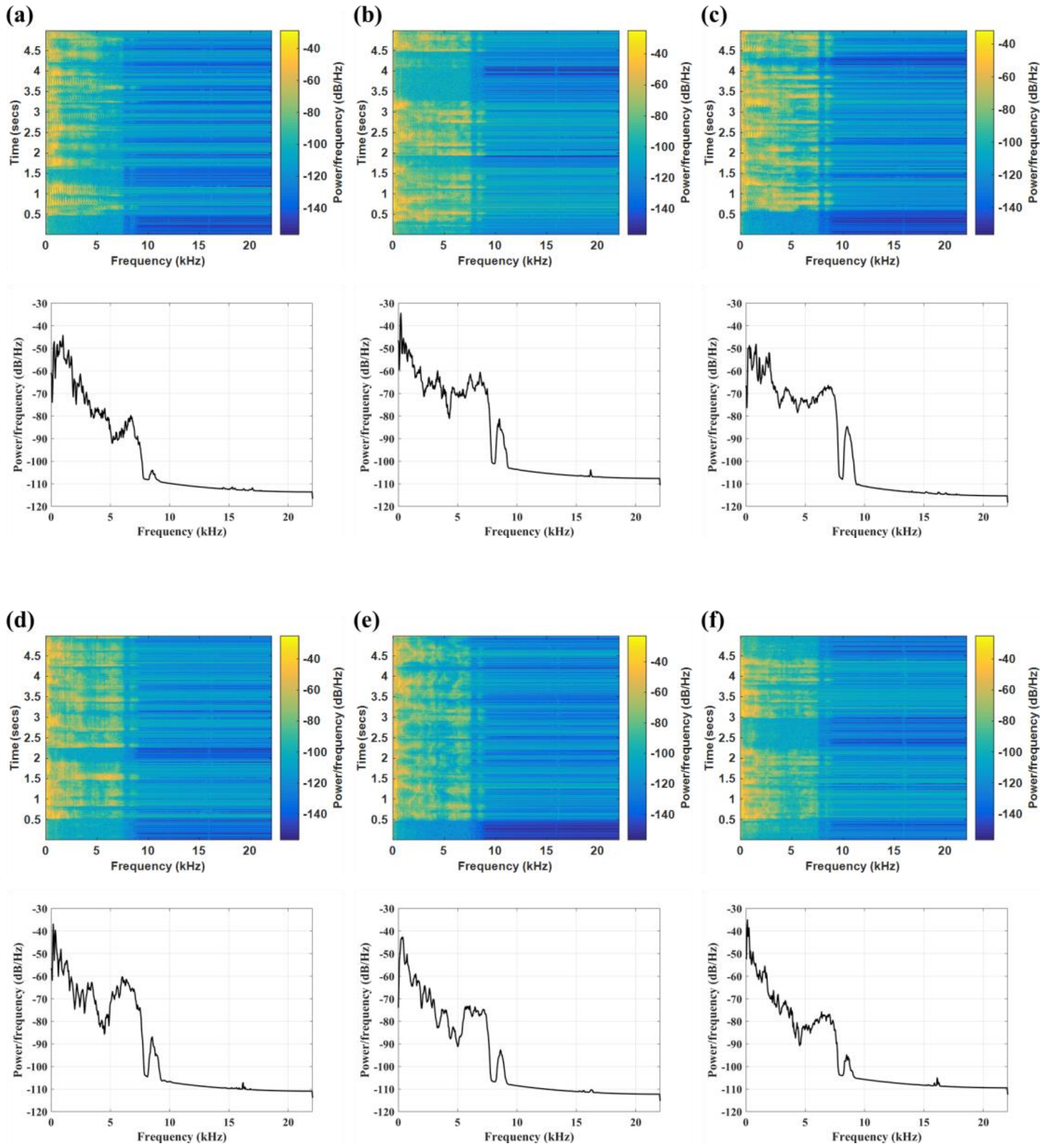

Figure S8. Spectrogram (top) and power spectral density (bottom) of signals often heard in the speech, including (a)-(c) female speech and (d)-(f) male speech.

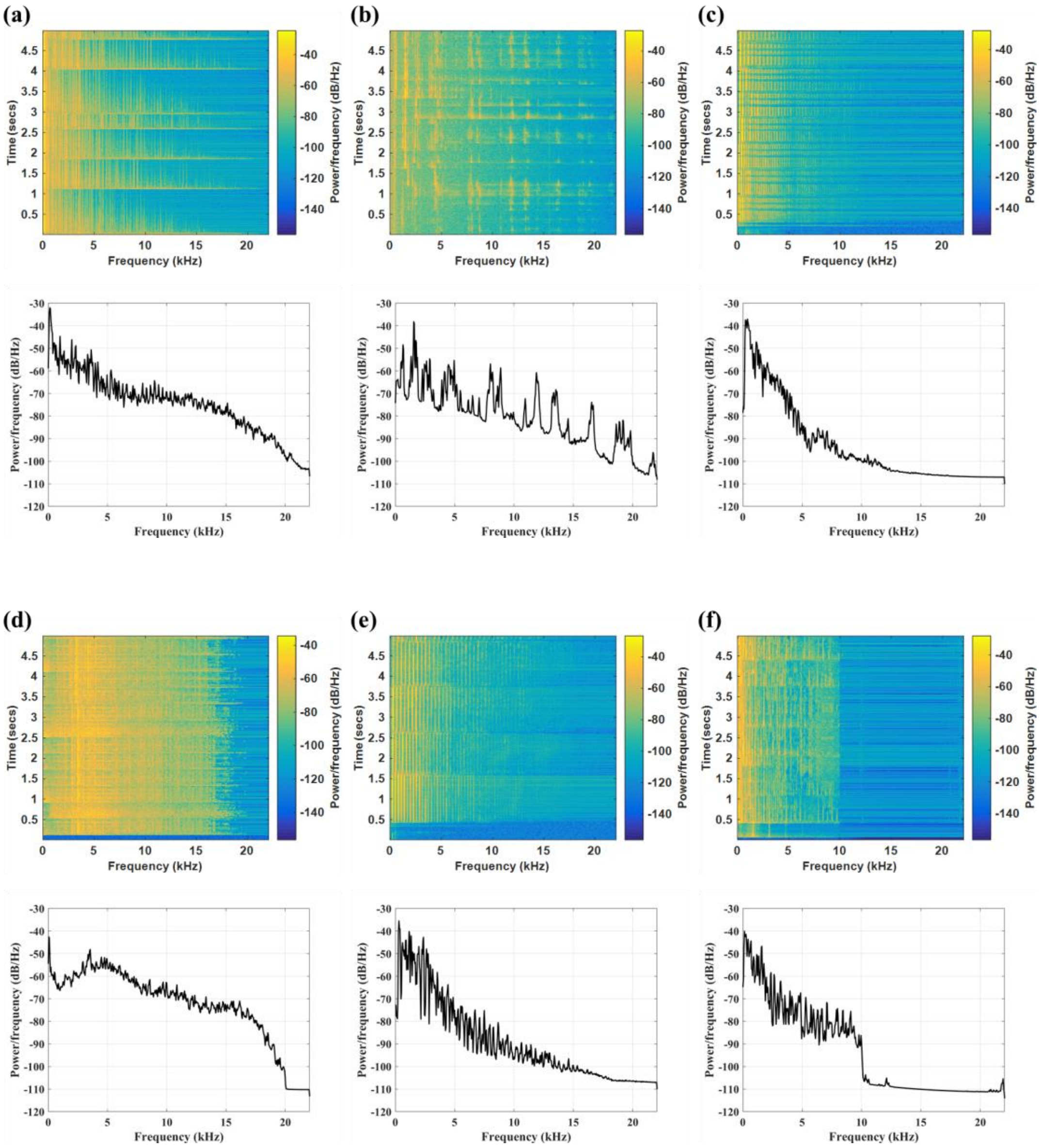

Figure S9. Spectrogram (top) and power spectral density (bottom) of six types of signals often heard in the concert, including (a) guitar, (b) chime, (c) saxophone, (d)drum, (e) violin and (f) Chinese folk music.

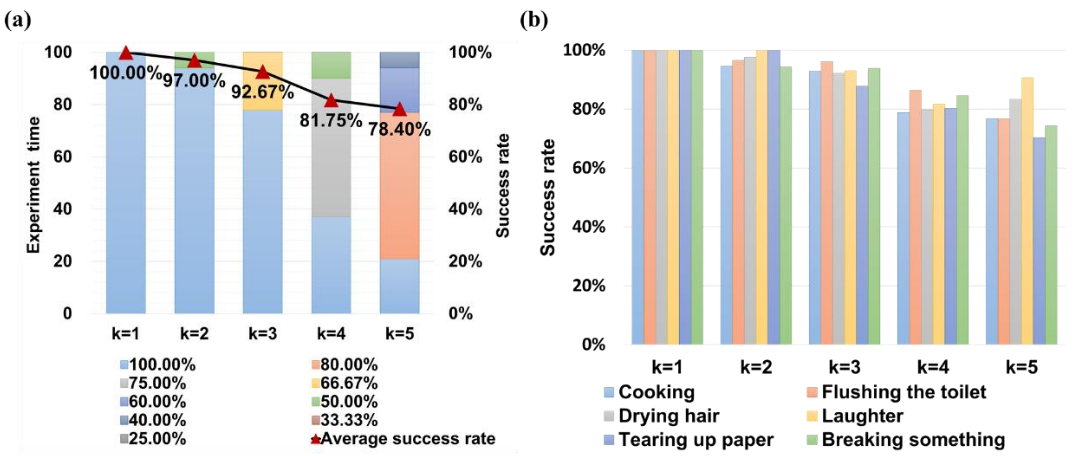

Figure S10. The results of the listening tests in the scenario of home organized by (a) the number of activated sources and (b) the audio contents.

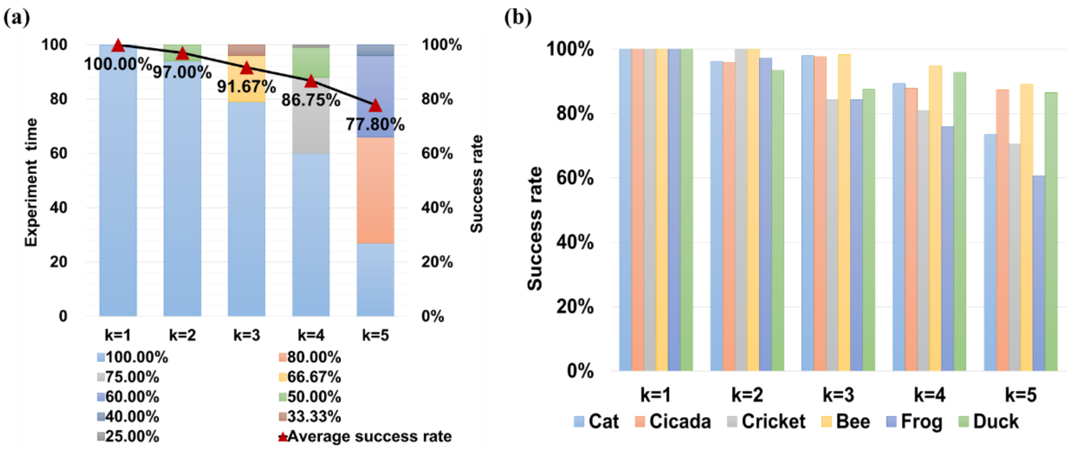

Figure S11. The results of the listening tests in the scenario of an animal farm organized by (a) the number of activated sources and (b) the audio contents.

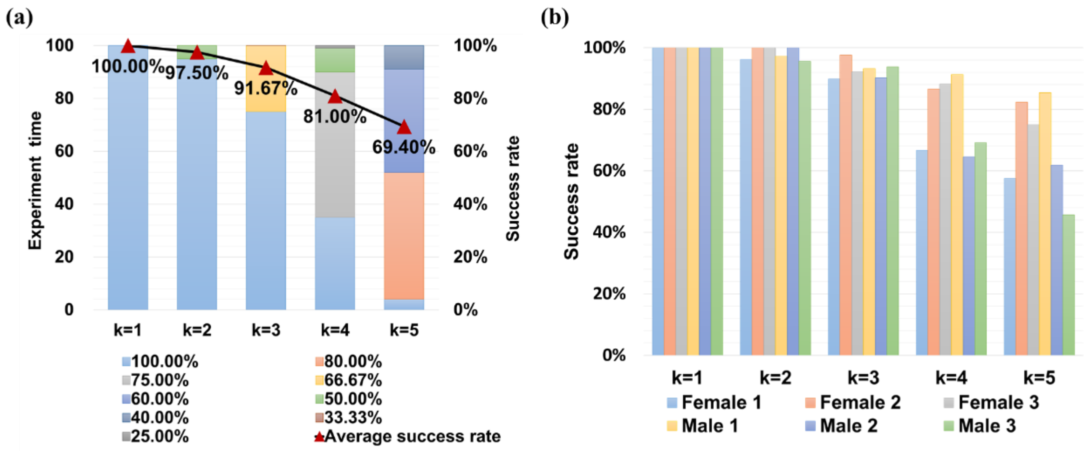

Figure S12. The results of the listening tests in the scenario of a speech organized by (a) the number of activated sources and (b) the audio contents.

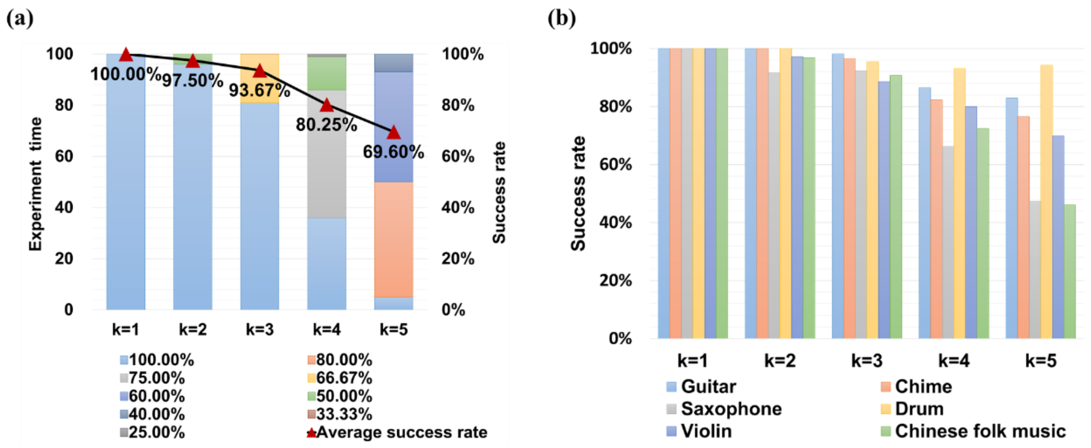

Figure S13. The results of the listening tests in the scenario of a concert organized by (a) the number of activated sources and (b) the audio contents.

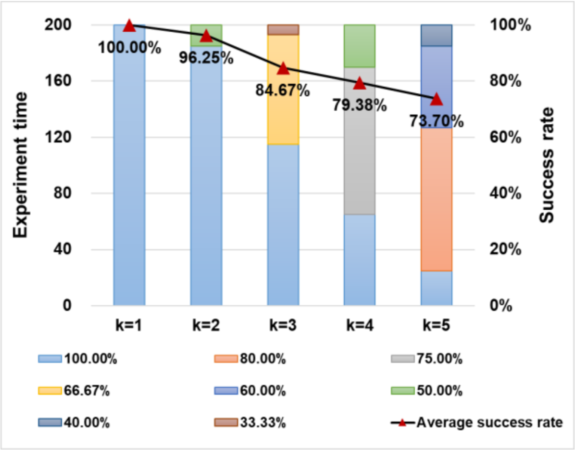

Figure S14. The results of the listening tests based on the Speech Commands dataset.

References

[1] R. A. Horn, C. R. Johnson, *Matrix Analysis*, Cambridge University Press, **2012**.

[2] H. Hotelling, *J. Educ. Psychol.* **1933**, *24*, 417.
